# Development and feasibility of an automated call monitoring intervention for older wheelchair users: the MOvIT project

**DOI:** 10.1186/s12913-015-1048-0

**Published:** 2015-09-16

**Authors:** Claudine Auger, William C. Miller, Jeffrey W. Jutai, Robyn Tamblyn

**Affiliations:** Center for Interdisciplinary Research in Rehabilitation of Greater Montreal and School of Rehabilitation, Université de Montréal, C.P. 6128, succursale Centre-ville, Montreal, QC H3C 3J7 Canada; GF Strong Rehabilitation Center and Graduate Program in Rehabilitation Sciences, Department of Occupational Science & Occupational Therapy, University of British Columbia, T325-2211 Wesbrook Mall, Vancouver, BC V6T 2B5 Canada; Bruyère Research Institute and Interdisciplinary School of Health Sciences, University of Ottawa, 25 University Private, Ottawa, ON K1N 6N5 Canada; Clinical and Health Informatics Research Group, Department of Medicine, and Department of Epidemiology, Biostatistics and Occupational Health, Faculty of Medicine, McGill University, 1140 Pine Avenue West, Montreal, QC H3A 1A3 Canada

## Abstract

**Background:**

Recent advances in wheeled mobility technology are multiplying opportunities for community integration and improved quality of life. The mobility needs of older wheelchair users are particularly complex due to a constellation of chronic conditions and comorbidities that may compromise optimal use of the device. The purpose of the Mobility Outcomes via Information Technologies (MOvIT) project is to examine the feasibility of automated calls for the systematic monitoring for adverse outcomes associated with wheelchair use.

**Methods:**

A two-phase mixed methods approach was used. Phase I involved user-centered development and face validation of a monitoring questionnaire with end-users (seven wheelchair users and five healthcare providers). Phase II tested the feasibility of monitoring outcomes using automated calls to administer the MOvIT questionnaire 1 and 3 months after wheelchair delivery with a prospective cohort of older adults (50–84 years of age). When problems were identified, the computer monitoring system notified a clinical coordinator who followed up with respondents requiring interventions. Feasibility data were extracted from the web database and from individual interviews covering perceived ease of use, usefulness and intention to use the MOvIT questionnaire in the future.

**Results:**

The MOvIT monitoring questionnaire developed in phase I tracks nine potential wheelchair-related adverse outcomes considered important for end-users: 1) non-use of wheelchair, 2) pain, 3) skin condition, 4) positioning, 5) wheelchair incidents, 6) psychosocial issues, 7) restricted wheelchair participation, 8) limited wheelchair skills and knowledge, and 9) technical problems. In phase II, 92 individuals who received a wheelchair were eligible, 71 out of 92 accepted (77 %) and 65 out of 71 (92 %) completed the 3-month follow-up. In the sample of 65 participants, a wheelchair-related adverse outcome was confirmed by a rehabilitation professional for 58.5 %, and at least one recommendation was given to 66.2 % during the 3-month monitoring period. A majority of participants found the intervention useful (82.8 %) and said they intended to use the MOvIT monitoring questionnaire in the future (81.5 %). Participants made suggestions to make the calls more adaptive to various ability profiles.

**Conclusions:**

Automated calls tailored for individuals with mobility limitations and associated comorbidities are a promising approach to reach clients who need post-rehabilitation support.

## Background

Worldwide, it is estimated that 65 million people need manual or power wheelchairs (WCs) [1]. Older adults are four times more likely to rely on WCs [2], and their mobility needs are more complex due to a constellation of chronic conditions and comorbidities in comparison to younger adults [3, 4]. For example, after 50 years of age the sudden onset of disability presents a higher risk for restriction in social roles [5], and the persistence of restriction for community participation, particularly mobility outside the home, increases with age [6]. To insure a proper match between the device and the user, the importance of systematic follow-up after wheeled mobility device provision has been acknowledged by international agencies [1]. A timely follow-up allows the detection of adverse outcomes such as early stage pressure sores [7, 8], pain associated with WC use [9, 10], or WC-related incidents due to malfunction of the device [11]. However, reviews of the wheeled mobility outcome literature reveal that adverse outcomes are rarely monitored or addressed [12, 13], and available studies tend to focus on one problem at a time [14–16]. Collecting routine follow-up data encompassing multiple WC-related issues would be important and novel since healthcare teams rarely know if they are meeting their clients’ mobility needs beyond WC delivery [17–20].

Major obstacles to follow-up are the burden of assessment and limited human resources for data collection and interpretation of the results. One creative solution is the use of automated calls administered by an interactive voice response system (IVRS). An IVRS allows a computer to detect voice and/or keypad input, can ask prerecorded questions and generate audio messages through automated calls. Although the IVRS is connected to a complex electronic web-based system, the end-user accesses it with a standard telephone. Besides simplicity of use, this information technology may offer significant advantages in terms of staff workload [21] and costs [22, 23]. Thus the monitoring of WC users with an IVRS could allow rehabilitation centers to systematically follow their clients with a simple technology and ensure timely interventions only for those at higher risk during the critical months after WC prescription.

The Mobility Outcomes via Information Technologies (MOvIT) project uses an IVRS to detect older adults at risk of poor outcomes after a WC prescription and sends an electronic alert to a rehabilitation professional if needed. This paper reports on the feasibility of the MOvIT monitoring system, including the development of a questionnaire, and the early detection of adverse outcomes associated with WC use for adults above 50 years of age.

## Methods

The study involved a mixed methods two-phase approach. The goal of Phase 1 was the development and face validation of the MOvIT monitoring questionnaire, while Phase 2 evaluated the feasibility of using an IVRS to administer the MOvIT questionnaire with a prospective cohort of WC users 1 and 3 months after WC delivery. The project was approved by the institutional review board of the Center for Interdisciplinary Research in Rehabilitation of Greater Montreal (CRIR-559-1110) and written informed consent was obtained from each participant.

### Phase I Development and face validation of the screening questionnaire

We followed the approach suggested by Streiner and Norman [24] to develop the experimental version of the questionnaire: 1) preliminary conceptual decisions, 2) item generation and response scaling, and 3) face validation.

#### Preliminary conceptual decisions

The Taxonomy of Assistive Technology Device Outcomes [25] suggests generic classes of outcomes as the most pertinent for the conceptual modeling of assistive technology device intervention–outcome relationships. We used the taxonomy to operationalize our screening tool relative to the three dimensions for determining assistive technology outcome effectiveness, subjective well-being, and social significance. The outcomes were extracted from literature reviews conducted on wheeled mobility outcomes, and studies reporting on wheeled mobility adverse outcomes shown in Table 1. The specific definitions of each dimension are also provided in Table 1.

**Table 1:** Conceptual framework of the monitoring questionnaire

Dimensions from taxonomy of assistive technology device outcomes	WC-related adverse outcomes tracked by the questionnaire	Source
EFFECTIVENESS: effect of assistive technology on domains of user functioning (ICF body functions, activity and participation) and effect of external influences on functioning and disability (ICF contextual factors)		
Body functions		
	-Pain/discomfort	Literature [9, 10, 50]
	-Skin problem	Literature [50–53]
	-Positioning problem	Literature [42, 50, 54]
	-WC incidents/accidents	Literature [15, 43, 45, 46, 50]
Activity and participation		
	-Limited WC skills and knowledge	Literature [50, 55, 56]/End-users
	-Restricted WC participation	Literature [13, 57, 58]
Environmental factors		
	-Reasons for non-use (weather conditions, home accessibility, transportation issues)	Literature [58]/End-users
SUBJECTIVE WELL-BEING: includes users’ cognitive and affective evaluations of how assistive technology has affected their lives		
Psychological functioning		
	-Psychosocial distress	Literature [28–31, 59]/End-users
Satisfaction		
	-Device dissatisfaction	Literature [32, 33, 60–62]/End-users
SOCIAL SIGNIFICANCE: extent to which outcomes are important to society, primarily in terms of their economic effect		
Device use		
	-Frequency of device use in various environments	Literature [59, 63–65]/End-users
Service use		
	-Device malfunction	Literature [16, 43, 44]/End-users

#### Item generation and response scaling

The MOvIT monitoring questionnaire was structured as an algorithm with a two-step process: 1) primary questions (called filter questions) assessed if a problem was present, and 2) secondary questions (called optional questions) were administered when an adverse outcome was detected to probe if the problem was related to use of the new WC. The questions were worded to elicit a nominal response, in order to allow automated administration methods with an IVRS using voice recognition or keypad numeric entry on the phone.

For each outcome identified by the literature review, preliminary items were created or adapted from published questionnaires when available. For the effectiveness dimension, the Seating Intervention Tool (SIT) provided the preliminary pool of items to cover body functions as it was designed for the case detection of WC seating needs by nursing staff with a broad focus on aspects such as pressure, discomfort, positioning, stability, as well as mobility. Similarly, the social significance dimension items covering frequency of device use were based on the environmental continuum of the Life-Space Assessment. Items related to the other constructs were derived from the literature review as shown in Table 1 and developed specifically for the MOvIT preliminary questionnaire.

#### Face validation of the MOvIT questionnaire

##### Participants

We assessed the face validity of the preliminary questionnaire filter and optional questions with end-users (WC users and healthcare providers). The target population of WC users was composed of adults 50 years of age and older who had received a manual or power WC in the previous year. Individuals living in long-term care facilities were excluded as the IVRS requires a direct telephone line. The healthcare providers had expertise in geriatric care and/or a minimum of three years of clinical practice, including at least one year at a WC clinic.

##### Procedures

Semi-structured individual interviews were conducted with the end-users. To establish rapport, we collected information on background and experience with WCs, including use and adverse outcomes. We then presented them with a mock-up version of the IVRS questionnaire, using audio files to simulate an automated call based on the script of the call algorithm. We administered the same mock-up version of the questionnaire during the first wave of six interviews, and revised iterations for the second wave of six interviews. The interviewer probed with questions based on cognitive processing theory. End-users commented on their thoughts while answering the questionnaire using think-aloud techniques. To facilitate the process, we presented suggestions made by previous participants to stimulate new ideas regarding the refinement of the items. Participants were asked to propose new formulations for questions if needed.

### Phase II Feasibility

#### Design

To assess the feasibility of the MOvIT monitoring intervention for the early detection of WC-related adverse outcomes, a prospective cohort of 65 WC users were followed at 3 time points: T0 (WC delivery), T1 (1 month post) and T2 (3 months post).

#### Participants

The target population was comprised of adults between the ages of 50 and 90 years who were prescribed a new manual or power WC. Individuals who were not living in the community one month after WC delivery and those who were unable to answer questions by voice or keypad were excluded.

#### MOvIT Monitoring questionnaire

**Fig. 1:**
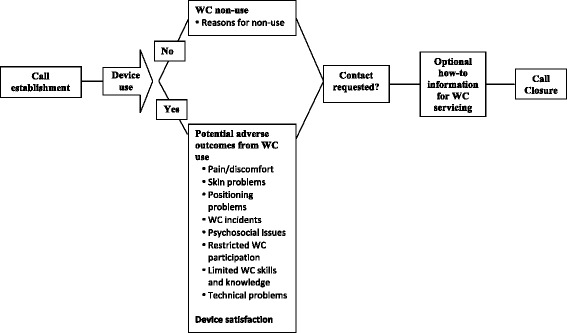
Call procedure

The final MOvIT monitoring questionnaire covers nine potential adverse outcomes: 1) non-use of WC, 2) pain, 3) skin problems, 4) positioning problems, 5) WC incidents, 6) psychosocial issues, 7) restricted WC participation, 8) limited WC skills and knowledge, and 9) technical problems (Appendix 1). The questionnaire has 16 general filter questions, and 22 optional questions to probe about the severity or link with WC use. Each item comprises decision rules programmed by an IVRS provider (TelAsk) in the IVRS to determine the presence of potential WC-related adverse outcomes. The call procedure (illustrated in Fig. 1) starts with three filter questions about WC use in different environments during the preceding month. If the individual did not use the device during the month preceding the assessment, the script proceeds with a series of questions about reasons for non-use (e.g. architectural barriers or transportation issues). If the individual used the WC during the preceding month, a series of 10 filter questions about potential adverse outcomes related to WC use, 2 filter questions about psychosocial distress, and one filter question on satisfaction (5-level response scale) are administered. If no WC-related adverse outcomes are detected, respondents have the option to request to be contacted by the staff if they have other concerns. An optional menu providing WC repair procedure information through phone, email or mail is available at the end of the T2 call.

#### Procedures

We contacted participants prior to their appointment for WC delivery at the rehabilitation center. A period of 2 weeks before or after WC delivery was tolerated to complete the baseline assessment. At baseline, the research coordinator tested an automated call to familiarize the participant with use of the keypad and voice recognition. Participants selected the preferred day of the week and time for their calls. The system was programmed to call at 1 month (T1) and 3 months (T2) post-WC delivery to administer the MOvIT questionnaire. It made 2 to 12 attempts over 4 days to reach the participant according to rules for a busy signal, no answer, answering machine, hang-up or partial call.

When a response to MOvIT items indicated there was an adverse outcome in one or more of the 9 domains assessed, a clinical coordinator (occupational therapist) from each site was notified electronically to call the subject back. This call back consultation occurred within seven working days. The coordinator accessed a web interface to see the call results and baseline information. Upon completion of the call, the coordinator confirmed each adverse outcome identified by the automated call and entered a summary note in the web-based record with clinical hypotheses about likely cause(s) and recommendations.

#### Semi-structured questionnaire

Individual interviews led by the first author (CA) were conducted with all participants after completion of the T2 call. The questionnaire started with a general open-ended question about the WC users’ experience with the MOvIT automated calls, followed by probes covering perceived ease of use (duration, clarity, speed, technical issues, input mode), usefulness and intention to use MOvIT monitoring in the future based on the Post-Study System Usability Questionnaire.

#### Feasibility

To assess feasibility we extracted data from the MOvIT database concerning call duration, call completion rate, number of cases requiring a callback, frequency and types of adverse outcomes detected by the automated call, frequency and types of adverse outcomes confirmed by the clinical coordinator, and frequency and types of recommendations.

#### Wheelchair user characteristics

Family income, highest level of education completed, age, sex, diagnosis, device type (manual vs power WC), and experience with WC use (first vs renewal WC) were collected at baseline.

### Analyses

#### Phase I Development and face validation of the monitoring questionnaire

An iterative approach was used to refine the preliminary questionnaire. The analysis was split into two waves of six participants. The results from the first wave of interviews were analyzed and implemented in a revised questionnaire. During the second wave of interviews, we edited the questionnaire after each iteration until we obtained data replication or redundancy [26] regarding the questionnaire content (e.g. item formulation, additional items), as well as the best process for the monitoring intervention (e.g. timing of the calls).

#### Phase II Feasibility

Quantitative analyses consisted of descriptive statistics (means, SD, and/or proportions). Acceptance rate was inferred from the ratio of eligible participants who agreed to participate, and attrition rate was the proportion of enrolled participants who were lost to follow-up because they were unable to complete at least one automated call or the T2 interview. To identify items that triggered unnecessary call backs, we calculated the ratio of WC-related adverse outcomes confirmed by the clinical coordinator versus detected by the automated calls. The characteristics of participants *vs*. non-participants were compared using t-tests and an alpha of *p* < 0.05.

To develop a better understanding concerning perceived ease of use of the automated calls and usefulness, the responses to the semi-structured questionnaire were coded, regrouped into themes and occurrences were counted. Most responses were grouped along a 5-point continuum ranging from clearly unproblematic to clearly problematic. Usefulness was categorized as useful for self, others, self and others or not useful. Intention to use was dichotomized (yes or no). Summary statistics were used to present these data.

## Results

### Phase I Development and face validation of the screening questionnaire

Twelve end-users participated in validating the questionnaire: 4 men and 3 women WC users aged 52 to 80 years of age who had 1.5 months to 20 years of experience with manual or power WCs; 4 occupational therapists and 1 orthotist who had 9.5 to 25 years of experience with WC prescription and the geriatric population.

Table 1 shows the conceptual framework that was used to structure the development and assist with the face validation of the MOvIT monitoring questionnaire. Two types of changes were made to the preliminary questionnaire: 1) item modifications, and 2) item additions. An example of item modification consisted of modifying the conceptual definition of the environment from a continuum (using the WC in the home, neighborhood, town) to a dichotomous definition (Q5/Q6, inside/outside home) to better reflect the vocabulary commonly used by end-users. Moreover, modifications to four optional items were requested by healthcare providers to insure that cases flagged as positive would present a severe problem or a significant worsening of a pre-existing condition (Q7b, Q7d, Q15, Q17). Their concern was to avoid unnecessary call backs about pre-existing problems or other problems unrelated to use of the new WC.

The item additions were either co-constructed with end-users or selected from available questionnaires. Six new items were co-constructed with end-users, namely: use of the device during transportation (Q11), weather reasons for non-use (Q8), mechanical breakdown and deficiencies (Q20), caregiver issues (Q26), and detailed “how-to” information about WC servicing (Q21, Q39). The suggestion to add items about psychosocial issues came from WC users. Nine items (Q27-Q35) are from the Patient Health Questionnaire three-item version (PHQ-3) [27] since a simplified version for older adults was available [28] and was validated with WC users [29–31]. However, these psychosocial items required a second layer of analysis by the clinical coordinator during the telephone contact to determine if the psychosocial distress was caused by the WC or not. Finally, WC users asked to end the questionnaire on a positive note when no adverse outcomes were detected using a general satisfaction question (Q36) adapted from the Quebec User Evaluation of Satisfaction with Assistive Technology [32].

All items use a dichotomous response scale (yes or no), except for the general satisfaction question Q36, which is adapted to provide a 5-level response scale ranging from not satisfied at all to very satisfied [32] (Appendix I).

### Phase II Feasibility

**Fig. 2:**
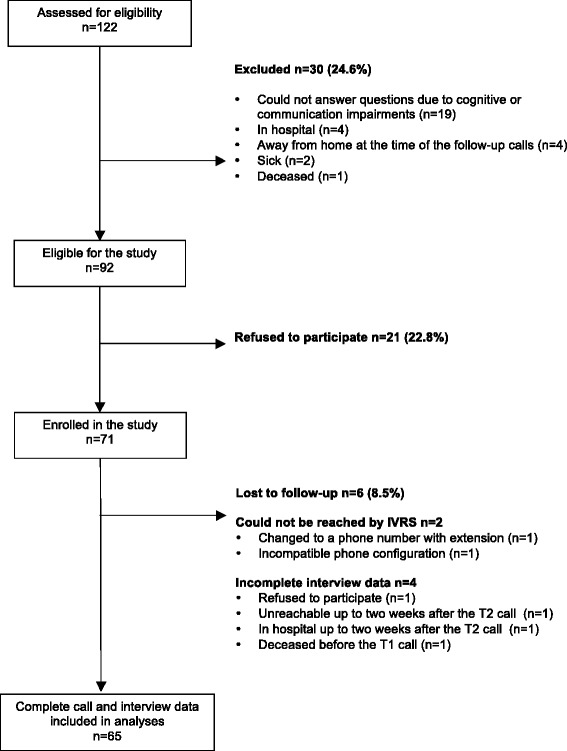
Feasibility study flow

A total of 122 individuals were contacted; 92 were eligible, 71 were enrolled, and 65 completed the call schedule and final interview, resulting in a 77 % (71/92) acceptance rate and 92 % (65/71) completion rate. The reasons for exclusion and loss to follow-up are listed in Fig. 2 and show that the main reason for exclusion was the presence of cognitive-communication impairments. The median (SD) duration of the call was 7.0 (1.9) and 8.0 (1.7) minutes at T1 and T2 respectively.

The sample was composed of 36 women and 29 men with a mean age (SD) of 68.7 years (9.5). Approximately two-thirds were prescribed a manual WC (*n* = 42; 64.6 %) and one-third a power WC (*n* = 23; 35.4 %). A first WC was obtained by 63.1 % of the sample (*n* = 25 manual WCs; *n* = 16 power WCs) while 36.9 % got a renewal WC (*n* = 17 manual WCs; *n* = 7 power WCs). A majority of the participants had neurological conditions (*n* = 50; 76.9 %), followed by musculoskeletal (*n* = 13; 20 %) and medically complex conditions (*n* = 2; 3.1 %). Other impairments affected fine motor (*n* = 25; 38.5 %), speech (*n* = 15; 23.1 % dysarthria or aphasia) and sensory functions (*n* = 6; 9.2 %; *n* = 1 legal blindness and *n* = 5 auditory impairments). Participants enrolled (*n* = 71) did not differ significantly from those with complete data (*n* = 65) with regard to sex, age, device use and health conditions (*t*-test *p* > 0.16).

Table 2 reports the WC-related adverse outcomes identified by the automated calls and confirmed by the screening intervention of the clinical coordinator at T1 and T2. Out of 65 participants, 69.2 % (*n* = 45) required contact at one point and at least one problem was confirmed for 58.5 % (*n* = 38) of the sample. A total of 96 problems were identified by the automated call and 78 of them were confirmed as WC-related adverse outcomes by the clinical coordinator. The potential WC-related adverse outcomes tracked by the screening questionnaire were each identified in 2 to 26 participants during the first 3 months of monitoring. Other reasons for calling back were 15 (23.1 %) participants who reported low satisfaction and 6 (9.2 %) contacts requested by participants.

**Table 2:** WC-related adverse outcomes based on calls and screening by clinical coordinator (*N* = 65)

	Detected by IVRS call				Confirmed by clinical coordinator
	T1 only	T2 only	T1 and T2	Total	Total
	n	n	n	n (%)	n (%)
Any WC-related adverse outcome	10	11	24	45 (69.2)	38 (58.5)
1-Non-use	0	1	1	2 (3.1)	1 (1.5)
2-Pain/discomfort	6	2	5	13 (20.0)	13 (20.0)
3-Skin problem	1	1	0	2 (3.1)	1 (1.5)
4-Positioning problem	3	3	5	11 (16.9)	10 (15.4)
5-WC incident	3	2	2	7 (10.8)	7 (10.8)
6-Psychological distress	7	5	1	13 (20.0)	2 (3.1)
7-Restricted WC participation	-	14	0	14 (21.5)	13 (20.0)
8-Limited WC skills/knowledge	3	3	2	8 (12.3)	8 (12.3)
9-Technical problems	5	15	6	26 (40.0)	23 (35.4)
Total	28	46	22	96	78
Not satisfied	7	3	5	15 (23.1)	15 (23.1)
Contact requested	5	1	0	6 (9.2)	6 (9.2)

Legend: T1: results for 1-month call exclusively; T2: results for 3-month call exclusively; T1 and T2: problem present at both calls; Total: problem present at 1-month or 3-month call

The most frequently confirmed adverse outcomes were technical issues with the WC (*n* = 23; 35.4 %), followed by restricted WC participation, such as having difficulty doing important activities inside or outside the home (*n* = 13; 20.0 %), pain and discomfort (*n* = 13; 20.0 %), and positioning problem (*n* = 10; 15.4 %). Some adverse outcomes were not confirmed by the clinical coordinators because the participant pressed yes by mistake. Other questions such as psychological distress items were not confirmed due to the questionnaire design. By design, these screening questions (Q27-Q35) required a second layer of analysis by the clinical coordinator to establish causality with use of the new WC, and thus 11 out of 13 flags for psychological distress were not confirmed because the distress was unrelated to WC use (e.g. mourning for a deceased family member).

We examined the type and frequency of recommendations made by the clinical coordinator to explore the usefulness of the T1 and T2 calls. We found that 66.2 % (*n* = 43) of study participants received at least one recommendation during the monitoring period. Out of a total of 54 recommendations given, the most frequent were for a clinical re-evaluation (*n* = 16; 29.6 %) or mechanical adjustment (*n* = 13; 24.1 %). Some overlap was observed between the recommendations of the two calls since a total of 16 (29.6 %) recommendations were given at T1 exclusively, 23 (42.6 %) at T2 exclusively, and 15 (27.8 %) were repeated between T1 and T2. The repeated recommendations were due to the need for another appointment to resolve the problem for 53.3 % (*n* = 8) or to non-compliance to return to the clinic for an intervention to address the identified item of concern for 46.7 % (*n* = 7).

When asked to tell us about their experience with the MOvIT automated calls (Table 3), many described how they felt about being monitored (e.g. cared for, respected for their opinion) or reported what they liked (e.g. call display) or disliked (e.g. slow pace of the script) about various aspects of the system. Although designed for WC user self-report, it is interesting to note that five caregivers answered as proxy respondents. The reasons provided were: participant was unavailable, caregiver was on a shared line in case participant needed help, physical help was given to click on the keypad responses but responses were provided by WC user, and inability to understand the questions.

**Table 3:** Results from semi-structured usability questionnaire

Question		Clearly unproblematic	More or less unproblematic	Neutral	More or less problematic	Clearly problematic
OVERALL EXPERIENCE		E.g. It was easy to use. It covers a lot of aspects.	E.g. It went well. As long as I hear well, it’s alright. Only one thing, my shoulder hurts after a while and I can’t hear with the other ear.		E.g. I had difficulty with some questions but I knew that someone would call back.	E.g. It’s long. It’s boring. It’s stupid. It makes no sense.
**Tell me about your experience with the automated calls for the follow-up of your new WC**						
	n (%)/65	32 (49.2)	10 (15.4)	12 (18.5)	7 (10.8)	4 (6.2)
DURATION		E.g. It did not last 5 min. If they had asked more questions, I would have answered more.	E.g. It was quite short. I think the first call was shorter than the second one.		E.g. It was a little long and the talking speed was slow… if it was a normal conversation, it would have taken half the time.	E.g. It was too long on my cell phone. I have to pay fees.
**What did you think about the length of the call?**						
	n (%)/65	37 (56.9)	3 (4.6)	8 (12.3)	13 (20.0)	4 (6.2)
CLARITY		E.g. The questions are very clear. The male and female voice … The man talks very well. The questions are asked clearly. They complement each other.	E.g. I understood what they asked. Sometimes I hesitated, but I did my best to answer.		E.g. Either the question is not clear or the response options are too limited, just yes or no. One question was missing response options.	E.g. I wondered if [the call] was made for persons with an intellectual disability. Short sentences and so clearly articulated as though they were afraid we wouldn’t understand.
**What did you think about the clarity of the call, with respect to the type or tone of voice?**						
	n (%)/65	51 (78.5)	4 (6.2)	0 (0)	7 (10.8)	3 (4.6)
SPEED		E.g. They gave us enough time. It was perfect.	E.g. Would be easier in my mother tongue.		E.g. It was a little fast.	E.g. Very very long. Too long. Some people may need such a slow pace. There should be a slow and a fast option.
**What did you think about the speed of the call, such as the speed of the questions, or the time you had to answer?**						
	n (%)/65	44 (67.7)	1 (1.5)	6 (9.2)	9 (13.8)	5 (7.7)
TECHNICAL ISSUES		E.g. No. When I wasn’t here, they called the next day.	E.g. They called me back the next day because I had pressed the wrong button.		E.g. I didn’t hear my name on the second call.	E.g. They stopped the call.
**Can you describe any technical problems you experienced during the automated calls?**						
	n (%)/65	48 (73.8)	3 (4.6)	3 (4.6)	7 (10.8)	4 (6.2)
INPUT MODE		E.g. I used the keypad. Never had to repeat.	E.g. When I answered with yes or no, they asked me to repeat. I pressed with my finger instead.		E.g. I didn’t answer fast enough, that’s why they had to repeat the questions.	E.g. This was the main problem. They never understood me. The machine did not work properly I think.
**Can you explain how you proceeded to answer the call (voice or keypad)? Did you have to repeat your responses?**						
	n (%)/65	43 (66.2)	16 (24.6)	0 (0)	4 (6.2)	2 (3.1)
USEFULNESS		**Useful for me**	**Useful for me and others**	**Useful for others**	**Not useful**	
**Do you think that these calls were useful? Please describe why.**		E.g. I figured that they care for me. If something had gone wrong, they would have called me.	E.g. If it makes a change to my chair, yes. It will make wheelchairs better. There’s not only me, there’s others.	E.g. It’s good for the person who gives the chair. She knows everything is ok with the chair.	E.g. Not useful. Not useless. It did not change anything in my life.	
	n (%)/64	32 (50.0)	11 (17.2)	10 (15.6)	11 (17.2)	
INTENTION TO USE		**Yes**		**I don’t know**	**No**	
**If your rehabilitation center offered this monitoring service in the future, would you register?**		E.g. I will always participate because it helps you, it helps me and it helps everyone.			E.g. No, I would rather call if I have a problem.	
	n (%)/65	53 (81.5)		3 (4.6)	9 (13.8)	

The automated call was not problematic for a majority of participants with respect to call duration (*n* = 48; 73.8 %), clarity (*n* = 55; 84.7 %), and speed (*n* = 51; 78.5 %). When probed about call duration, some participants mentioned that the call was a little long or too long (*n* = 17; 26.2 %), while others (*n* = 5; 7.7 %) felt that the slower pace of the dialogue caused this impression. Clarity and speed were linked since many who felt that the pace of the call was appropriate also appreciated script clarity, while a few (*n* = 5; 7.7 %) found that the clarity of the pronunciation was exaggerated. Some wished to speed up by skipping the directions or using the rapid pace option (*n* = 4; 6.2 %), while others said they needed more time to think prior to answering (*n* = 3; 4.6 %).

Technical issues with the automated calls were clearly unproblematic for most participants (*n* = 48; 73.8 %), and a majority were able to answer without any repetition (*n* = 43; 66.2 %). When asked, four (6.1 %) participants reported major technical problems experienced during the calls: one had a temporary disturbance with her telephone line due to reconfiguration of the intercom system in her building while three experienced voice recognition problems that led to call disconnection.

When asked about the usefulness of the monitoring intervention all participants except one had an opinion. A majority (*n* = 53/64; 82.8 %) felt it was useful. Participants reported it was useful for them personally (*n* = 32; 50.0 %), useful for both themselves and others (*n* = 11; 17.2 %), or useful for other WC users or the healthcare team (*n* = 10; 15.6 %). The reasons provided by those who thought the intervention was not useful were that no changes were required for them personally (*n* = 8; 12.5 %), they preferred to call their occupational therapist directly when they had a problem (*n* = 2; 3.1 %), or their WC-related adverse outcome was unresolved (*n* = 1; 1.6 %). When asked if they would register if offered this monitoring service in the future, 81.5 % (*n* = 53/65) of the participants reported interest in using such a service.

## Discussion

The aim of this study was to develop a questionnaire for use in a phone monitoring intervention for the early detection of adverse outcomes associated with WC use and to assess its feasibility for community-living middle-aged and older adults. The questionnaire monitors nine potential adverse outcomes using automated calls and telephone consultations by occupational therapists. The results suggest it was feasible to implement the monitoring intervention as approximately 77.2 % of eligible individuals from three WC clinics participated and 92.0 % of the enrolled participants completed the protocol. A large majority of WC users (82.8 %) thought it would be a useful follow-up tool.

Lack of follow-up after prescription of a mobility device is one of the key complaints of WC users [33, 34]. There are many obstacles to the implementation of a systematic follow-up, including the poor applicability of available measures for clinical use, the geographic dispersion of users, and restricted access to post-discharge rehabilitation services [35, 36]. Feasibility is a major obstacle when attempting to integrate systematic data collections from WC users in real life clinical practices [19]. Most existing outcome measures for follow-up after WC prescription are in-depth assessments that measure single constructs such as device satisfaction [32], psychosocial impact [37], or mobility [38], and they can take up to 30 min each to administer. The MOvIT questionnaire could be used as a preliminary step in identifying those who need further follow-up or in-depth assessments. A distinctive contribution of the data collection approach we are proposing is its potential compatibility with alternative data collection methods, such as automated calls through interactive voice response systems, telephone interviews by clerical staff, or online assessments. The ultimate goals are to remove the burden from busy clinical personnel, maximize the early detection of poor WC-related outcomes to alert agencies about unmet needs, prevent the recurrence of WC-related adverse events, and relieve the healthcare system of a tremendous financial burden.

Developing the questionnaire for the systematic monitoring of community-living adults involved several challenging conceptual and methodological issues. Our first challenge was to define and select undesirable results, negative consequences or injuries resulting from WC use. Reviews of adverse outcomes are quite rare and account for only 4 % of all systematic reviews in health care [39]. Moreover, Golder at al. [39] found that these reviews typically focus on prespecified effects, which limits their scope. Thus we had to base our initial selection of WC-related adverse outcomes on published reviews that used a broad search approach. Using the Taxonomy of Assistive Technology Device Outcomes [25] to anchor these concepts proved helpful as there is no existing framework of WC-related adverse outcomes. Subsequently, the input of end-users refined our questions to include the most relevant problems that are amenable to intervention. End-users, particularly healthcare providers, insisted on detecting adverse outcomes that are clinically relevant (e.g. worsening of a pre-existing condition directly caused by the new WC) and could be ameliorable following a screening intervention (e.g. resolved by modifying the WC configuration). We encountered a challenge in trying to reconcile the suggestions made by WC users and healthcare providers, particularly around the construct of psychological distress. These questions were requested by WC users in Phase I while most healthcare providers did not feel it was part of their mandate to address psychological issues. Results of Phase II showed that only 2 out of 13 flags for general psychological distress were confirmed as WC-related. The next iterations of MOvIT will have to find the right balance to address the need for psychosocial support without adding an unnecessary burden on clinical coordinators. Finally, it is very important to focus on elements that are considered severe or high-priority and most modifiable by the target population because they are more likely to lead to behavioral change [40]. Including clinicians is recognized as an essential step to promote knowledge transfer and facilitate adoption of an innovation in clinical practice [41]. We engaged them throughout the monitoring tool development process, which may have contributed to tailoring it to their needs and to raising its overall acceptability and potential for implementation in the future.

Regarding the usefulness of the monitoring tool for early detection of WC-related adverse outcomes, we believe the 1- and 3-month calls were both necessary as some unique adverse outcomes and recommendations were identified at each time point. Our findings indicate similar proportions of WC-related adverse outcomes as reported in previous studies. For instance, the rate of pain/discomfort and positioning complaints/issues that we found (15-20 %) is comparable to the 22 % reported by Bourbonnière et al. [42]. Our estimate of a 35 % rate of technical problems is also in line with past studies reporting breakdown rates in the range of 33-45 % [16, 43, 44]. Our WC incident rate of 11 % is lower than in other publications, primarily because these studies spanned one to three years of WC use with rates of 31-38 % for falls and 14-47 % for WC injuries [15, 43, 45, 46]. The proportion of respondents who were dissatisfied with their WC in our study was also lower than for previous studies reporting after up to 18 months of WC use (23 % *vs*. 34 % [47]) but higher than for studies that did not control for duration of WC use (23 % *vs*. 11 % [48]).

Finding the right balance between respondent burden and accommodating the wide range of capacities and comorbidities present in various diagnoses in the population served by WC clinics remains challenging. As proposed by Miller et al. [49], one promising option is the concept of a self-adjusting IVRS that would automatically adapt to the respondent’s cognitive and sensory capabilities when the system has to repeat instructions. Our participants asked for options such as pause, repeat or speed adjustment. We also believe that alternative formats should be developed in the future to offer the possibility of choosing between IVRS, on-line and proxy-respondent versions with the results centralized in a single database. This would allow each individual to select the most compatible modality, as a ‘one size fits all’ approach may not be possible with a heterogeneous population.

The results of this feasibility study should be interpreted with caution because the study was designed to explore the potential utility of an IVRS for monitoring assistive technology. The enrolments took place over a short period of time between October 2011 and January 2012, meaning that most of the follow-up calls took place during the winter. Participants repeatedly mentioned winter weather as an obstacle to returning for a servicing appointment, and this should be taken into account in future studies by comparing seasonal variation in the rates of unresolved issues. A seasonal effect may explain why a fair percentage (*n* = 24; 36.9 %) of the sample had unresolved problems at both 1 and 3 months post-WC delivery, as some delayed their servicing visits until spring. The representativeness of the very old age group (85+ years) is also limited, possibly due to a higher prevalence of cognitive-communication impairments in this group. At the present stage, it is not possible to make inferences about the overall effectiveness of the intervention in reducing adverse events or costs following WC prescription because the study had a relatively small sample size and was not designed to address this question. However our results show that a majority of WC users accepted the technology and found it easy to use, which justifies investigating the effect of the intervention in a larger randomized controlled trial.

## Conclusions

In this study we developed an intervention with end-users to monitor nine potential WC-related adverse outcomes using information technologies. It was feasible to implement the intervention in WC clinics using an IVRS connected to a web interface, and a large majority of WC users thought it would be a useful follow-up tool. Tracking adverse outcomes soon after WC prescription is a crucial aspect to give providers feedback about the needs of specific individuals, and also to identify gaps at a higher system-level. We believe that the monitoring tool will simplify that task for clinical teams.

## Abbreviations

IVRS: Interactive voice response system
WC: Wheelchair
